# Single-cell immunophenotyping of the fetal immune response to maternal SARS-CoV-2 infection in late gestation

**DOI:** 10.21203/rs.3.rs-311000/v1

**Published:** 2021-03-16

**Authors:** Juan Matute, Benjamin Finander, David Pepin, Xinbin Ai, Neal Smith, Jonathan Li, Andrea Edlow, Alexandra Villani, Paul Lerou, Brian Kalish

**Affiliations:** Massachusetts General Hospital and Harvard Medical School; Boston Children’s Hospital and Harvard Medical School; Massachusetts General Hospital; Massachusetts General Hospital and Harvard Medical School; Massachusetts General Hospital and Harvard Medical School; Harvard Medical School; Massachusetts General Hospital and Harvard Medical School; Harvard University; Massachusetts General Hospital and Harvard Medical School; The Hospital for Sick Children and University of Toronto

**Keywords:** COVID19, SARS-CoV-2, pregnancy, cord blood mononuclear cells

## Abstract

During the COVID-19 pandemic, thousands of pregnant women have been infected with SARS-CoV-2. The implications of maternal SARS-CoV-2 infection on fetal and childhood well-being are unknown. We aimed to characterize the fetal immune response to maternal SARS-CoV-2 infection. We performed single-cell RNA sequencing and T-cell receptor (TCR) sequencing on cord blood mononuclear cells (CBMC) from newborns of mothers infected with SARS-CoV-2 in the third-trimester (cases) or without SARS-CoV-2 infection. We identified widespread gene expression changes in CBMC from cases, including upregulation of interferon-stimulated genes and Major Histocompatibility Complex genes in CD14 + monocytes; transcriptional changes suggestive of activation of plasmacytoid dendritic cells, and activation and exhaustion of NK cells and CD8 + T-cells. Lastly, we observed fetal TCR repertoire expansion in cases. As none of the infants were infected with SARS-CoV-2, our results suggest that SARS-CoV-2 maternal infection might modulate the fetal immune system in the absence of vertical transmission.

## BRIEF SUMMARY

SARS-CoV-2 infection in the third-trimester of pregnancy without vertical transmission promotes widespread gene expression changes and TCR repertoire clonal expansion in cord blood leukocytes.

## Introduction

Millions of people worldwide have or will become infected with SARS-CoV-2, causing Coronavirus Disease 2019 (COVID-19), and the infection of pregnant women with SARS-CoV-2 infection has been widespread^1–6^. Despite the prevalence of antepartum infection, we have a limited understanding of the implications of SARS-CoV-2 infection on maternal, fetal, and offspring health. To date, there are limited case reports of vertical, mother-to-child transmission of SARS-CoV-2^7–12^, and vertical transmission remains rare in most pregnancies complicated by maternal SARS-CoV-2 infection^1,6,13–17^. Nonetheless, in the absence of direct fetal infection and toxicity, maternal SARS-CoV-2 infection may still affect fetal development. Maternal immune activation during pregnancy after viral infection without vertical transmission can have long-term consequences for the newborn, including abnormal neurologic^18,19^ or immune system development^20^.

Pregnancy is a complex and precarious immunologic state, and there is no data on the effect of SARS-CoV-2-dysregulated immune state during pregnancy on the fetus. The related SARS-CoV epidemic in 2003 was linked to high rates of spontaneous abortions, preterm birth, and intrauterine growth restriction^21^. Given the number of pregnant women infected with SARS-CoV-2 worldwide, it is important to determine the potential transgenerational implications of infection with SARS-CoV-2 during pregnancy beyond vertical transmission. To date, research on the implications of SARS-CoV-2 infection during pregnancy on the offspring immune system has been limited to postnatal evaluation of infants born to mothers infected with SARS-CoV-2 during pregnancy without a non-exposed control group^22,23^, which may be also confounded by *ex-utero* determinants of immune development during the first week of life^24^. in the present study, we characterize the composition and cell type-specific transcriptional landscape of umbilical cord blood mononuclear cells (CBMC) from term gestation infants (>37 weeks) born to mothers infected with SARS-CoV-2 in the third trimester without vertical transmission. This immunogenomic investigation provides evidence of both innate and adaptive fetal immune transcriptional changes in pregnancies complicated by SARS-CoV-2 infection. Our results suggest that even in the absence of vertical transmission, SARS-CoV-2 maternal infection in the third trimester might modulate the fetal immune system.

## Results And Discussion

To characterize the fetal immunologic landscape in pregnancies complicated by maternal SARS-CoV-2 infection, we performed droplet-based single-cell RNA sequencing (scRNAseq) of CBMC from infants born to mothers with SARS-CoV-2 infection during pregnancy (cases) and infants born to mothers without SARS-CoV-2 infection (controls). CBMCs from three cases and three controls were obtained from our biorepository^25^. None of the three infants in this study born to mothers with SARS-CoV-2 were positive for SARS-CoV-2 postnatally, had detectable SARS-CoV-2 mRNA in placenta or developed any neonatal morbidity. All mothers with COVID19 in the third trimester were classified as having mild disease without respiratory support^26^. Infants born to mothers negative for SARS-CoV-2 and asymptomatic (universal screening at admission for labor) during the same epoch served as controls. Maternal comorbidities were matched between cases and controls as feasible. Table 1 displays demographic and clinical data from the cases and controls.

CBMCs were processed on the 10X Genomics Single-Cell Immune platform (see Methods). After quality control and doublet removal, we included 14,748 cells with high quality single-cell transcriptomes from cases and 11,222 cells from controls in our dataset. (See quality control metrics in Supplementary Fig. 1A–B). The cellular population composition was visualized using uniform manifold approximation and projection (UMAF, Fig. 1A), and cell types were inferred by cluster-specific canonical marker genes (Fig. 1B-C). We did not observe any differences in cell cluster composition between cases and controls (Supplementary Fig. 1C).

To explore transcriptional signatures in fetal immune cells associated with maternal SARS-CoV-2 infection, we performed differential gene expression (DGE) analysis within cell types comparing cases and controls. Genes with a false discovery rate (FDR) < 5% were considered statistically significant. We identified hundreds of genes across nearly all cell types with altered expression (Fig. 2A). We used gene ontology (GO) analysis to broadly classify genes significantly disrupted by maternal SARS-CoV-2 infection based on DGE (Supplemental Table 1).

CD14 + monocytes were grouped into 5 clusters and CD16 + monocytes were grouped into one cluster (Fig. 2B). CD14 +sub-populations demonstrated variable expression of inflammatory genes, including *ACSL1, ADGRE2, CD300E and PADI4,* which aligns with prior single cell analysis showing monocyte diversity^27^. Consistent with data from adult COVID-19 patients^28,29^, we found that CD14 + monocytes from cases demonstrated increased expression of ISG (Fig. 2C) and concomitant *IFNAR2* down regulation (Supplemental Fig. 2a), which could reflect exposure to interferon prenatally^30^. GO analysis of DGE in CD14 + monocytes demonstrated enrichment of genes associated with antigen presentation and viral translational termination and reinitiation (Fig. 2D). Cord blood (CB) CD14 + monocytes from cases also showed upregulation of major histocompatibility class (MHC) I and II genes suggesting activation in response to interferon signaling^31^. Furthermore, CD14 +monocytes from cases showed upregulation of TLR receptor transcripts (*TLR2, TLR4* and *TLR5*) paired with upregulation of *FOS and* downregulation of transcriptional inhibitors of NFKB (*NFKBIA* and *NFKB/E*), all of which are associated with increased NFKB activation and cytokine production^32^ (Supplemental Fig. 2A). Of note, CD14 +monocytes from cases had decreased expression of autophagy (*ATG14, ATG2A, ATG3*) and endoplasmic reticulum stress (*XBP1, HSPA5*) genes, which may contribute to a defect in macrophage differentiation^33^ (Supplemental Fig. 2A).

Similar to CD14 + monocytes, we identified induction of ISG in non-classical CB monocytes (CD16+) (Fig. 2C). In contrast to CD14 + monocytes, we found that there was decreased expression of cell adhesion genes (including *PLAUR* and *THBS1*), attenuation of immune activation signaling pathways genes (*FOS, FOSB, MAP3K8, STAT6,* and *FCER1G*), and decreased expression of inflammatory molecules like resistin (*RETN*) (Supplemental Fig. 2B). Together, these results suggest induction of ISG in monocytes from cases compared to controls and differences in transcriptional changes in classical and non-classical monocytes that might suggest preferential activation of classical monocytes in cases compared to controls.

We captured the transcriptomes of both plasmacytoid and conventional dendritic cells (pDC and cDC, respectively) in CB. In adults infected with SARS-CoV-2, both types of DCs are functionally impaired, and there is an increased ratio of cDCs to pDCs in severe patients^34^. In our study, CB cDC from cases showed increased expression of ISG like *IFITM3* and *APOBEC3A* (Fig. 2E). Transcription factor zinc finger E box–binding homeobox 2 (Zeb2) plays a crucial role in promoting cDC and pDC development by downregulating Inhibitor of DNA binding protein 2 (ID2)^35,36^. *ZEB2* was increased in cDC and *ID2* was decreased in pDC from CB of cases, which might be evidence of a shift towards pDC in CB from infants exposed to SARS-COV-2 *in utero* (Fig. 2E). Fetal cDC from cases showed a transcriptional profile suggestive of innate immune activation including increased expression of *PIK3CB,* which is downstream of TLR5 and TLR7^37^, as well as increased transcription of *CCL5,* which can be upregulated after TLR3 stimulation (Fig. 2E)^38^. Evidence of impaired cDC maturation was suggested by upregulation of *ID1,* which antagonizes dendritic cell differentiation and antitumor immunity in mice^39^, as well as increased *MAFB* transcription, which suppresses cDC maturation^40^. cDCs from cases also demonstrated decreased expression of *FOSB* and many MHC II genes^41^. pDCs in cases also showed markers of immune activation, including upregulation of *RELB,* which promotes DC activation through RelB-p50 dimer^42^, upregulation of MHC Class I and Class II genes, and UPR activation, as shown by increased transcription of *XBP1*^43^ (Supplemental Fig. 2C). Together, these transcriptional findings could be consistent with activation of pDC over cDC in the CB of cases, potentially through activation of TLRs.

In adults, SARS-CoV-2 infection is associated with fewer blood NK cells but a higher activation state in circulating NK cells^44^. We identified two clusters of cord blood NK cells. One population of NK cells (cluster 1) expressed higher levels of *GZMB,* while the second population of NK cells (cluster 2) expressed *IL7R* and *XCL1,* suggesting that cluster 1 corresponded to CD56dim and cluster 2 corresponded to CD56bright NK cells, as *NCAM1* (CD56) is technically not well captured in scRNAseq^45^. Similar to adult NK cells, CB NK cells from SARS-CoV-2-positive pregnancies showed signs of exposure to interferon, including induction of ISG genes like *IFI6, IFIT2* and *IRF9* (Fig. 2F)^44,46,47^ We identified increased transcription of *CCL4,* expression of cytotoxic genes including *GNLY, GZMA, GZMB and GZMH,* and increased transcription of *IFNG,* paired with decreased expression of NK inhibitory molecules (Fig. 2F)^44,46,47^. There were transcriptional changes associated with exhaustion, such as decreased expression of *KLRG1* and *SIGLEC7*^48^. DGE in NK cells between cases and controls were enriched for genes related to the interferon-alpha response, regulation of NK cell cytokine production, and viral transcription (Fig. 2G).

In adults with acute COVID19, there is a heterogeneous adaptive immune response in peripheral blood, including B-cell receptor and T-cell receptor arrangements specific to SARS-CoV-2^46^. Given these findings, we evaluated whether maternal infection with SARS-CoV-2 had any effect on CB lymphocyte gene expression. We were able to identify three clusters of CB B-cells (Fig. 1A,B). We also identified three clusters of T-cells (Fig. 3A). Cluster 1 corresponded to CD8 + T-cells. Cluster 2 and 3 corresponded to helper T cells^49^. Increased expression of *CCR7* in T-cell Cluster 2 suggests this cluster includes either naive T-cells or central memory T-Cells^50^ and increased *CTSW* and *KLRB1* in Cluster 3 suggest this cluster includes effector and memory T-cells^51,52^.

In B cells from infants exposed to SARS-CoV-2 in utero, we identified 3 clusters of CB B-cells corresponding to non-plasma (Cluster 1 and 2) and plasma cells (Cluster 3) based on *MZB1* expression^46^ (Supplementary Fig. 2D). In B-cells from infants born to mothers infected with SARS-CoV-2, we identified decreased markers of B-cell receptor activation in all clusters. Specifically, we found decreased transcription of *NR4A1, CD69* and *CD83* in all B-cells (Supplemental Fig. 2E). *NR4A1* encodes Nur77, an orphan nuclear receptor, that is induced upon B-cell activation in peripheral blood in humans^53^. *CD83* is expressed upon activation of B-cells, and activated T-cells induce CD83 on B-cells via CD40 engagement^54^. Concordant with decreased B-cell activation, we also found downregulation of *CD69*^55^, decreased expression AP-1 and NFAT genes^56^, and decreased expression of anti-apoptotic genes, including *BCL2 and BCL2A1*^57^ (Supplemental Fig. 2E). Transcriptional changes suggestive of potential B-cell dysfunction, combined with decreased transplacental transmission of IgG against SARS-CoV-2 compared to IgG against other antigens^6,58^, might translate into potential impairments in antibody mediated immunity to SARS-CoV-2 in neonates born to mothers with COVID19.

In adults with COVID19, CD8 + T-cells show decreased cytotoxic potential and exhaustion driven by IL-6^59^. Similar to adults, CB CD8 + T-cells from cases demonstrated transcriptional signatures suggestive of impaired cytotoxic activity, including decreased expression of *GZMA*^60^, and CD8 T-cell exhaustion, including downregulation of *FOS*(Fig. 3B)^61^. Furthermore, there was increased expression of genes associated with central memory T-cells, including *KLRB1*^52^ and *CCR7*^62^, that might be associated with fetal CD8 activation with SARS-CoV-2 (Fig. 3B). GO analysis of DGEs in CD8 + T-cells demonstrated enrichment for genes associated with T-cell tolerance, proliferation, and the response to interferon gamma (Supplemental Fig. 3A). In T-cell Cluster 2, we found increased expression of IL6-IL17 axis genes including *RORA, ARID5A, RBPJ,* and *IL6ST* in cases compared to controls (Supplemental Fig. 3B). IL6-IL17 axis has been implicated in mediating the neurodevelopmental effects of maternal immune activation in mice^63^.

T-cell antigen receptor (TCR) repertoire in T-cells reflects selection by self and foreign antigens. To investigate the repertoire of TCRs in CB from SARS-CoV-2-exposed pregnancies and controls, we performed single cell TCR sequencing. A total of 1,943 T-cells were analyzed, and T-cells with TCR information were well equally distributed between subject and T-cell populations (Supplemental Fig. 3C and 3D). Clonal expansion was significantly increased in T-cells from pregnancies complicated by maternal SARS-CoV-2 infection, with 40.4% of T-cells having > 5 clones in the cases, compared with 30.9% in the controls (Kolmogorov-Smirnov Test p value 2.2e-16) (Fig. 3D). The T-cell clonal expansion in CB from cases is consistent with results of T-cell repertoire analysis from adults infected with SARS-CoV-2^46^.

Despite the novelty of scRNAseq analysis of CBMC, our study is exploratory and has several limitations. Importantly, the small number of samples limits the generalizability of our conclusions. However, few studies have evaluated CB immune populations by single-cell transcriptomics^64,65^, and our results illustrate an important and potentially underrecognized population in the COVID-19 pandemic that should be further studied. All cases included in this study were classified as mild maternal SARS-CoV-2 infection; more severe maternal infection could result in more dramatic or different fetal immune genomic signatures. Furthermore, the time from infection to delivery and cord blood collection likely affects the immune phenotype observed in cord blood. As the time of maternal infection and birth in our cohort fluctuates between 7 and 66 days, more pronounced findings could be found with samples with a more consistent timing between infection and collection. Lastly, all mothers affected with SARS-CoV-2 in our cohort had comorbidities including well-controlled thyroid dysfunction, obesity or gestational diabetes. Although we included mothers with similar comorbidities in the control population (except for gestational diabetes) and all these comorbidities were medically managed, it is possible that our results are influenced by the comorbidities of the mothers. However, thyroid disease, obesity or gestational diabetes in the mother have not been reported to trigger the transcriptional response patterns we observed in cases compared to controls^66–69^.

Despite these limitations, the present study identifies transcriptional changes suggestive of a fetal immune response after maternal infection with SARS-CoV-2 in the absence of vertical transmission and suggest potential trans-placental immune implications of maternal SARS-CoV-2 infection beyond vertical transmission. The source of signals promoting transcriptional changes in neonatal monocytes and other immune cells in the absence of vertical transmission is unknown. *Ex-vivo* studies have shown that transplacental transfer of IL1, IL6 and TNF does not occur^70^. Type I interferons are increased in peripheral circulation of patients with mild COVID19^28^, but the ability of interferon to cross the human placenta is unclear^71^. Our results raise the possibility that pro-inflammatory signaling in the mother in response to SARS-CoV-2 might promote interferon signaling at the feto-maternal interface. Further experimental data needs to be collected to clarify how maternal infection with SARS-CoV-2 influences the fetal immune system. Given the extensive literature linking maternal immune dysregulation and abnormal fetal development in viral infections, this study raises important questions about untoward effects of maternal SARS-CoV-2 on the fetus, even in the absence of vertical transmission and highlights the need of further studies to better characterize the fetal immune response in pregnancies affected by SARS-CoV-2 infection.

## Methods

### Sample collection, cryopreseivation and placental viral load

The subjects were six infants born at term to mothers with or without SARS-CoV-2 infection in the third-trimester. Parents of the infants provided informed consent before sample collection and study participation. The study was approved by the Institutional Review Board of the Mass General Brigham (IRB 2020P001478 and IRB2020P000804). Cord mononuclear cells were collected using ficoll and cryopreserved as described^25^. We used DMSO as our cryopreservant agent as it adequately conserves gene expression profiles in cryopreserved cells compared to fresh cells in droplet-based single-cell RNA sequencing^72^. We excluded preterm infants, as a strong proinflammatory signature in CB has been reported in infants born preterm^73^. None of the infants were exposed to prenatal steroids, were diagnosed as IUGR, or had any neonatal morbidities. Placental viral load was measured as previously reported^74^.

### Single-cell RNA-sequencing

CBMC aliquots were thawed in a 37°C water bath and resuspended in RPMI-1640 with 10% FBS (Thermo Fisher). Samples were centrifuged at 350 x g for 7 minutes at 4°C. Cells were resuspended in 100 microliters of 1X PBS with 2.5% FBS and 2 mM EDTA.

Dead cells and red blood cells were depleted using the EasySep Dead Cell Depletion Kit and EasySep RBC Depletion Reagent (Stem Cell), according to manufacturer instructions. Cells were resuspended in RPMI/10% FBS and counted. Cells were loaded on to the 10X Chromium controller at a targeted recovery density of 10,000 cells per sample. Samples were processed and sequencing libraries were created using the 10X the Chromium Next GEM single-cell V(D)J Reagent Kit v1.1 with human TCR V(D)J Enrichment following manufacturer instructions.

### Single-cell RNA-sequencing data analysis

Sequencing data were aligned to the genome and processed using the 10X Genomics Cell Ranger software, version 4.0.0. All cells were combined into a single dataset. Doublets were removed using Scrublet version 0.2.1, and the remaining cells were re-clustered. Mitochondrial genes were filtered from the dataset. Cells with fewer than 250 or more than 2500 unique genes were excluded. Cells were then clustered using the Seurat R package (Version 3.2.3). Specifically, the SCT functionality of Seurat was used to identify cell types that did not depend upon unique aspects of individual samples. Clustering resolution was set to 0.8, and the first 15 principal components were used. The data were log normalized and scaled to 10,000 transcripts per cell. The expression of known marker genes was used to assign each cluster to one of the main cell types. The Seurat FindMarkers function was used to identify genetic markers of cellular subtypes.

### Identification of differentially expressed genes between cases and controls

To identify differentially expressed genes by cell type, we performed a differential gene expression analysis using Monocle2. The analysis was conducted on each cell type and also certain unions of cell types with common traits. The data were modeled and normalized using a negative binomial distribution, and counts data was normalized for gene length and read depth. Genes whose false discovery rate (FDR) was less than 5% were considered statistically significant. GO analysis was performed using gprofiler2 version 0.2.0, and terms were selected from the Biological Process category of GO terms.

### T cell receptor sequencing

TCR sequencing data was analyzed using the R package scRepertoire (Version 3.12).

## Supplementary Material

Supplement

Supplement

Supplement

Supplement

Supplement

Supplement

Supplement

## Figures and Tables

**TABLE 1:** Clinical characteristics of Cases and controls.

Subjects	Onset of Symptoms (GA)	SARS-CoV-2 PCR (GA)	Delivery (QA)	Days between onset of symptoms and birth	Maternal symptoms at test	Delivery Mode	Any Labor?	Maternal Comorbidities	Sex Assigned At Birth	Placental viral load by RT-PCR	24h nasopharyngeal viral load by RT-PCR
Control 1	NA	39.9	40	NA		VD	Yes		Male	1	NA
Control 2	NA	38.9	39.4	NA		CS	Yes	BMI > 30, Thyroid disease	Female	1	NA
Control 3	NA	38.4	38.6	NA		CS	Yes	BMI > 30	Female	ND	NA
Case 1	30.3	30.7	39.7	66	Fever/Chills, nasal congestion, Loss of taste/smell, sore throat, night sweats	VD	Yes	Thyroid disease	Male	1	Negative
Case 2	34.4	35.4	40.1	40	Cough, Fever/Chills, Myalgias, Headache, chest discomfort	CS	Yes	Diabetes/GDM, BMI > 30, Thyroid disease	Male	1	Negative
Case 3	39	39.4	40	7	Cough, Fever/Chills	VD	Yes	BMI > 30	Female	1	Negative

GA: Gestational Age, VD: Vaginal Delivery, CS: Cesarean section, ND: Not done, NA: Not applicable

## Data Availability

Sequencing data has been deposited in the Gene Expression Omnibus under accession GSE165193.

## References

[1] Chen L. Clinical Characteristics of Pregnant Women with Covid-19 in Wuhan, China. New Engl J Med 382, e100 (2020). 32302077 10.1056/NEJMc2009226 PMC7182016

[2] Prabhu M. Pregnancy and postpartum outcomes in a universally tested population for SARS-CoV-2 in New York City: a prospective cohort study. Bjog Int J Obstetrics Gynaecol 127, 1548–1556 (2020). 10.1111/1471-0528.16403 PMC7361728 32633022

[3] Sutton D., Fuchs K., D’Alton M. & Goffman D. Universal Screening for SARS-CoV-2 in Women Admitted for Delivery. New Engl J Med 382, 2163–2164 (2020). 32283004 10.1056/NEJMc2009316 PMC7175422

[4] Huntley B. J. F. Rates of Maternal and Perinatal Mortality and Vertical Transmission in Pregnancies Complicated by Severe Acute Respiratory Syndrome Coronavirus 2 (SARS-Co-V-2) Infection: A Systematic Review. Obstetrics Gynecol 136, 303–312 (2020). 10.1097/AOG.0000000000004010 32516273

[5] Ellington S. Characteristics of Women of Reproductive Age with Laboratory-Confirmed SARS-CoV-2 Infection by Pregnancy Status – United States, January 22-June 7, 2020. Morbidity Mortal Wkly Rep 69, 769–775 (2020). 10.15585/mmwr.mm6925a1 PMC7316319 32584795

[6] Edlow A. G. Assessment of Maternal and Neonatal SARS-CoV-2 Viral Load, Transplacental Antibody Transfer, and Placental Pathology in Pregnancies During the COVID-19 Pandemic. Jama Netw Open 3, e2030455 (2020). 33351086 10.1001/jamanetworkopen.2020.30455 PMC7756241

[7] Raschetti R. Synthesis and systematic review of reported neonatal SARS-CoV-2 infections. Nat Commun 11, 5164 (2020). 33060565 10.1038/s41467-020-18982-9 PMC7566441

[8] Kotlyar A. Vertical Transmission of COVID-19: A Systematic Review and Meta-analysis. Am J Obstet Gynecol (2020) doi:10.1016/j.ajog.2020.07.049 PMC8479509 34597604

[9] Vivanti A. J. Transplacental transmission of SARS-CoV-2 infection. Nat Commun 11, 3572 (2020). 32665677 10.1038/s41467-020-17436-6 PMC7360599

[10] Chen H. Clinical characteristics and intrauterine vertical transmission potential of COVID-19 infection in nine pregnant women: a retrospective review of medical records. Lancet 395, 809–815 (2020). 32151335 10.1016/S0140-6736(20)30360-3 PMC7159281

[11] Fenizia C. Analysis of SARS-CoV-2 vertical transmission during pregnancy. Nat Commun 11, 5128 (2020). 33046695 10.1038/s41467-020-18933-4 PMC7552412

[12] Dong L. Possible Vertical Transmission of SARS-CoV-2 From an Infected Mother to Her Newborn. Jama 323, 1846–1848 (2020). 32215581 10.1001/jama.2020.4621 PMC7099527

[13] Mascio D. D. Risk factors associated with adverse fetal outcomes in pregnancies affected by Coronavirus disease 2019 (COVID-19): a secondary analysis of the WAPM study on COVID-19. J Perinat Med 0, (2020). 10.1515/jpm-2020-0539 33470966

[14] Dumitriu D. Outcomes of Neonates Born to Mothers With Severe Acute Respiratory Syndrome Coronavirus 2 Infection at a Large Medical Center in New York City. Jama Pediatr 175, (2021). 10.1001/jamapediatrics.2020.4298 PMC7551222 33044493

[15] Walker K. Maternal transmission of SARS-COV-2 to the neonate, and possible routes for such transmission: a systematic review and critical analysis. Bjog Int J Obstetrics Gynaecol 127, 1324–1336 (2020). 10.1111/1471-0528.16362 PMC7323034 32531146

[16] Khoury R. Characteristics and Outcomes of 241 Births to Women With Severe Acute Respiratory Syndrome Coronavirus 2 (SARS-CoV-2) Infection at Five New York City Medical Centers. Obstetrics Gynecol 136, 273–282 (2020). 10.1097/AOG.0000000000004025 32555034

[17] Knight M. Characteristics and outcomes of pregnant women admitted to hospital with confirmed SARS-CoV-2 infection in UK: national population based cohort study. Bmj 369, m2107 (2020). 32513659 10.1136/bmj.m2107 PMC7277610

[18] Al-Haddad B. J. S. Long-term Risk of Neuropsychiatric Disease After Exposure to Infection In Utero. Jama Psychiat 76, 594 (2019). 10.1001/jamapsychiatry.2019.0029 PMC6551852 30840048

[19] Al-Haddad B. J. S. The Fetal Origins of Mental Illness. Am J Obstet Gynecol 221, 549–562 (2019). 31207234 10.1016/j.ajog.2019.06.013 PMC6889013

[20] Abu-Raya B., Kollmann T. R., Marchant A. & MacGillivray D. M. The Immune System of HIV-Exposed Uninfected Infants. Front Immunol 7, 383 (2016). 27733852 10.3389/fimmu.2016.00383 PMC5039172

[21] Wong S. F. Pregnancy and perinatal outcomes of women with severe acute respiratory syndrome. Am J Obstet Gynecol 191, 292–297 (2004). 15295381 10.1016/j.ajog.2003.11.019 PMC7137614

[22] Liu P The immunologic status of newborns born to SARS-CoV2-infected mothers in Wuhan, China. J Allergy Clin Immun 146, 101–109.e1 (2020). 32437740 10.1016/j.jaci.2020.04.038 PMC7211641

[23] Zeng H. Antibodies in Infants Born to Mothers With COVID-19 Pneumonia. Jama 323, 1848–1849 (2020). 32215589 10.1001/jama.2020.4861 PMC7099444

[24] Olin A. Stereotypic Immune System Development in Newborn Children. Cell 174, 1277–1292.e14 (2018). 30142345 10.1016/j.cell.2018.06.045 PMC6108833

[25] Shook L. L. Rapid establishment of a COVID-19 perinatal biorepository: early lessons from the first 100 women enrolled. Bmc Med Res Methodol 20, 215 (2020). 32842979 10.1186/s12874-020-01102-y PMC7447612

[26] NIH. COVID-19 Treatment Guidelines Panel. Coronavirus Disease 2019 (COVID-19) Treatment Guidelines. https://www.covid19treatmentguidelines.nih.gov/ (2020).

[27] Villani A.-C. Single-cell RNA-seq reveals new types of human blood dendritic cells, monocytes, and progenitors. Science 356, eaah4573 (2017). 28428369 10.1126/science.aah4573 PMC5775029

[28] Hadjadj J. Impaired type I interferon activity and inflammatory responses in severe COVID-19 patients. Science eabc6027 (2020) doi:10.1126/science.abc6027 PMC7402632 32661059

[29] Schulte-Schrepping J. Severe COVID-19 is marked by a dysregulated myeloid cell compartment. Cell 182, 1419–1440.e23 (2020). 32810438 10.1016/j.cell.2020.08.001 PMC7405822

[30] Arunachalam P S. Systems biological assessment of immunity to mild versus severe COVID-19 infection in humans. Science 369, 1210–1220 (2020). 32788292 10.1126/science.abc6261 PMC7665312

[31] KESKINEN P, RONNI T., MATIKAINEN S., LEHTONEN A. & JULKUNEN I. Regulation of HLA class I and II expression by interferons and influenza A virus in human peripheral blood mononuclear cells. Immunology 91, 421–429 (1997). 9301532 10.1046/j.1365-2567.1997.00258.x PMC1364012

[32] Dorrington M. G. & Fraser I. D. C. NF-κB Signaling in Macrophages: Dynamics, Crosstalk, and Signal Integration. Front Immunol 10, 705 (2019). 31024544 10.3389/fimmu.2019.00705 PMC6465568

[33] Germic N., Frangez Z., Yousefi S. & Simon H.-U. Regulation of the innate immune system by autophagy: monocytes, macrophages, dendritic cells and antigen presentation. Cell Death Differ 26, 715–727 (2019). 30737475 10.1038/s41418-019-0297-6 PMC6460400

[34] Zhou R. Acute SARS-CoV-2 infection impairs dendritic cell and T cell responses. Immunity 53, 864–877.e5 (2020). 32791036 10.1016/j.immuni.2020.07.026 PMC7402670

[35] Scott C. L. The transcription factor Zeb2 regulates development of conventional and plasmacytoid DCs by repressing Id2Zeb2 controls dendritic cell development. J Exp Medicine 213, 897–911 (2016). 10.1084/jem.20151715 PMC4886362 27185854

[36] Wu X. Transcription factor Zeb2 regulates commitment to plasmacytoid dendritic cell and monocyte fate. Proc National Acad Sci 113, 14775–14780 (2016). 10.1073/pnas.1611408114 PMC5187668 27930303

[37] Cavalieri D. DC-ATLAS: a systems biology resource to dissect receptor specific signal transduction in dendritic cells. Immunome Res 6, 10 (2010). 21092113 10.1186/1745-7580-6-10 PMC3000836

[38] Weinzierl A. O. Effective Chemokine Secretion by Dendritic Cells and Expansion of Cross-Presenting CD4−/CD8 + Dendritic Cells Define a Protective Phenotype in the Mouse Model of Coxsackievirus Myocarditis. J Virol 82, 8149–8160 (2008). 18550677 10.1128/JVI.00047-08 PMC2519575

[39] Papaspyridonos M. Id1 suppresses anti-tumour immune responses and promotes tumour progression by impairing myeloid cell maturation. Nat Commun 6, 6840 (2015). 25924227 10.1038/ncomms7840 PMC4423225

[40] Yang L., Li R., Xiang S. & Xiao W. MafB, a target of microRNA-155, regulates dendritic cell maturation. Open Life Sci 11, 46–54 (2016).

[41] Belz G. T. & Nutt S. L. Transcriptional programming of the dendritic cell network. Nat Rev Immunol 12, 101–113 (2012). 22273772 10.1038/nri3149

[42] Shih V. F.-S. Control of RelB during dendritic cell activation integrates canonical and noncanonical NF-κB pathways. Nat Immunol 13, 1162–1170 (2012). 23086447 10.1038/ni.2446 PMC3634611

[43] Beisel C. TLR7-mediated activation of XBP1 correlates with the IFNα production in humans. Cytokine 94, 55–58 (2017). 28408069 10.1016/j.cyto.2017.04.006

[44] Maucourant C. Natural killer cell immunotypes related to COVID-19 disease severity. Sci Immunol 5, eabd6832 (2020). 32826343 10.1126/sciimmunol.abd6832 PMC7665314

[45] Yang C. Heterogeneity of human bone marrow and blood natural killer cells defined by single-cell transcriptome. Nat Commun 10, 3931 (2019). 31477722 10.1038/s41467-019-11947-7 PMC6718415

[46] Zhang J.-Y. Single-cell landscape of immunological responses in patients with COVID-19. Nat Immunol 21, 1107–1118 (2020). 32788748 10.1038/s41590-020-0762-x

[47] Wilk A. J. A single-cell atlas of the peripheral immune response in patients with severe COVID-19. Nat Med 26, 1–7 (2020). 32514174 10.1038/s41591-020-0944-y PMC7382903

[48] Varchetta S. Unique immunological profile in patients with COVID-19. Cell Mol Immunol 1–9 (2020) doi:10.1038/s41423-020-00557-9 PMC7557230 33060840

[49] Ding J. Systematic comparison of single-cell and single-nucleus RNA-sequencing methods. Nat Biotechnol 1–10 (2020) doi:10.1038/s41587-020-0465-8 32341560 PMC7289686

[50] Campbell J. J. CCR7 Expression and Memory T Cell Diversity in Humans. JImmunol 166, 877–884 (2001). 11145663 10.4049/jimmunol.166.2.877

[51] Cano-Gamez E. Single-cell transcriptomics identifies an effectorness gradient shaping the response of CD4 + T cells to cytokines. Nat Commun 11, 1801 (2020). 32286271 10.1038/s41467-020-15543-y PMC7156481

[52] Fergusson J. R., Fleming V. M. & Klenerman P CD161-Expressing Human T Cells. Front Immunol 2, 36 (2011). 22566826 10.3389/fimmu.2011.00036 PMC3342360

[53] Ashouri J. F. & Weiss A. Endogenous Nur77 Is a Specific Indicator of Antigen Receptor Signaling in Human T and B Cells. J Immunol 198, 657–668 (2017). 27940659 10.4049/jimmunol.1601301 PMC5224971

[54] Kretschmer B., Kühl S., Fleischer B. & Breloer M. Activated T cells induce rapid CD83 expression on B cells by engagement of CD40. Immunol Lett 136, 221–227 (2011). 21277328 10.1016/j.imlet.2011.01.013

[55] Zimmermann M. Antigen Extraction and B Cell Activation Enable Identification of Rare Membrane Antigen Specific Human B Cells. Front Immunol 10, 829 (2019). 31040853 10.3389/fimmu.2019.00829 PMC6477023

[56] Gorter D. J. J. de, Vos J. C. M., Pals S. T. & Spaargaren M. The B Cell Antigen Receptor Controls AP-1 and NFAT Activity through Ras-Mediated Activation of Ral. J Immunol 178, 1405–1414 (2007). 17237388 10.4049/jimmunol.178.3.1405

[57] Slomp A. & Peperzak V. Role and Regulation of Pro-survival BCL-2 Proteins in Multiple Myeloma. Frontiers Oncol 8, 533 (2018). 10.3389/fonc.2018.00533 PMC6256118 30524962

[58] Atyeo C. Compromised SARS-CoV-2-specific placental antibody transfer. Cell (2020) doi:10.1016/j.cell.2020.12.027 PMC7755577 33476549

[59] Mazzoni A. Impaired immune cell cytotoxicity in severe COVID-19 is IL-6 dependent. J Clin Invest (2020) doi:10.1172/jci138554 PMC7456250 32463803

[60] Zhou Z. Granzyme A from cytotoxic lymphocytes cleaves GSDMB to trigger pyroptosis in target cells. Science 368, eaaz7548 (2020). 32299851 10.1126/science.aaz7548

[61] Wherry E. J. Molecular Signature of CD8 + T Cell Exhaustion during Chronic Viral Infection. Immunity 27, 670–684 (2007). 17950003 10.1016/j.immuni.2007.09.006

[62] Sallusto F., Lenig D., Förster R., Lipp M. & Lanzavecchia A. Two subsets of memory T lymphocytes with distinct homing potentials and effector functions. Nature 401, 708–712 (1999). 10537110 10.1038/44385

[63] Yockey L. J., Lucas C. & Iwasaki A. Contributions of maternal and fetal antiviral immunity in congenital disease. Science 368, 608–612 (2020). 32381717 10.1126/science.aaz1960

[64] Zhao Y. Single-cell transcriptomic landscape of nucleated cells in umbilical cord blood. Gigascience 8, (2019). 10.1093/gigascience/giz047 PMC6497034 31049560

[65] Jin X. Characterization of dendritic cell subtypes in human cord blood by single-cell sequencing. Biophysics Reports 5, 199–208 (2019).

[66] Mason E. Maternal Influences on the Transmission of Leukocyte Gene Expression Profiles in Population Samples from Brisbane, Australia. Plos One 5, e14479 (2010). 21217831 10.1371/journal.pone.0014479 PMC3013110

[67] Svensson J. Maternal Autoimmune Thyroid Disease and the Fetal Immune System. Exp Clin Endocr Diab 119, 445–450 (2011). 10.1055/s-0031-1279741 21667438

[68] Wilson R. M. Maternal obesity alters immune cell frequencies and responses in umbilical cord blood samples. Pediatr Allergy Immu 26, 344–351 (2015). 10.1111/pai.12387 PMC9271931 25858482

[69] Yanai S. Diabetic pregnancy activates the innate immune response through TLR5 or TLR1/2 on neonatal monocyte. J Reprod Immunol 117, 17–23 (2016). 27351455 10.1016/j.jri.2016.06.007

[70] Aaltonen R., Heikkinen T., Hakala K., Laine K. & Alanen A. Transfer of Proinflammatory Cytokines Across Term Placenta. Obstetrics Gynecol 106, 802–807 (2005). 10.1097/01.AOG.0000178750.84837.ed 16199639

[71] Yockey L. J. & Iwasaki A. Interferons and Proinflammatory Cytokines in Pregnancy and Fetal Development. Immunity 49, 397–412 (2018). 30231982 10.1016/j.immuni.2018.07.017 PMC6152841

[72] Wohnhaas C. T. DMSO cryopreservation is the method of choice to preserve cells for droplet-based single-cell RNA sequencing. Sei Rep-uk 9, 10699 (2019). 10.1038/s41598-019-46932-z PMC6650608 31337793

[73] Matoba N. Differential Patterns of 27 Cord Blood Immune Biomarkers Across Gestational Age. Pediatrics 123, 1320–1328 (2009). 19403498 10.1542/peds.2008-1222

[74] Fajnzylber J. SARS-CoV-2 viral load is associated with increased disease severity and mortality. Nat Commun 11, 5493 (2020). 33127906 10.1038/s41467-020-19057-5 PMC7603483

