# Supplementary material for: Single-cell immunophenotyping of the fetal immune response to maternal SARS-CoV-2 infection in late gestation
Source: Res Sq. 2021 Mar 16:rs.3.rs-311000. Preprint. [Version 1] doi: 10.21203/rs.3.rs-311000/v1 (PMC7987103; doi:10.21203/rs.3.rs-311000/v1)
Supplement: Supplement [file 3c7a1513e84db9e5c42688e3.pdf]

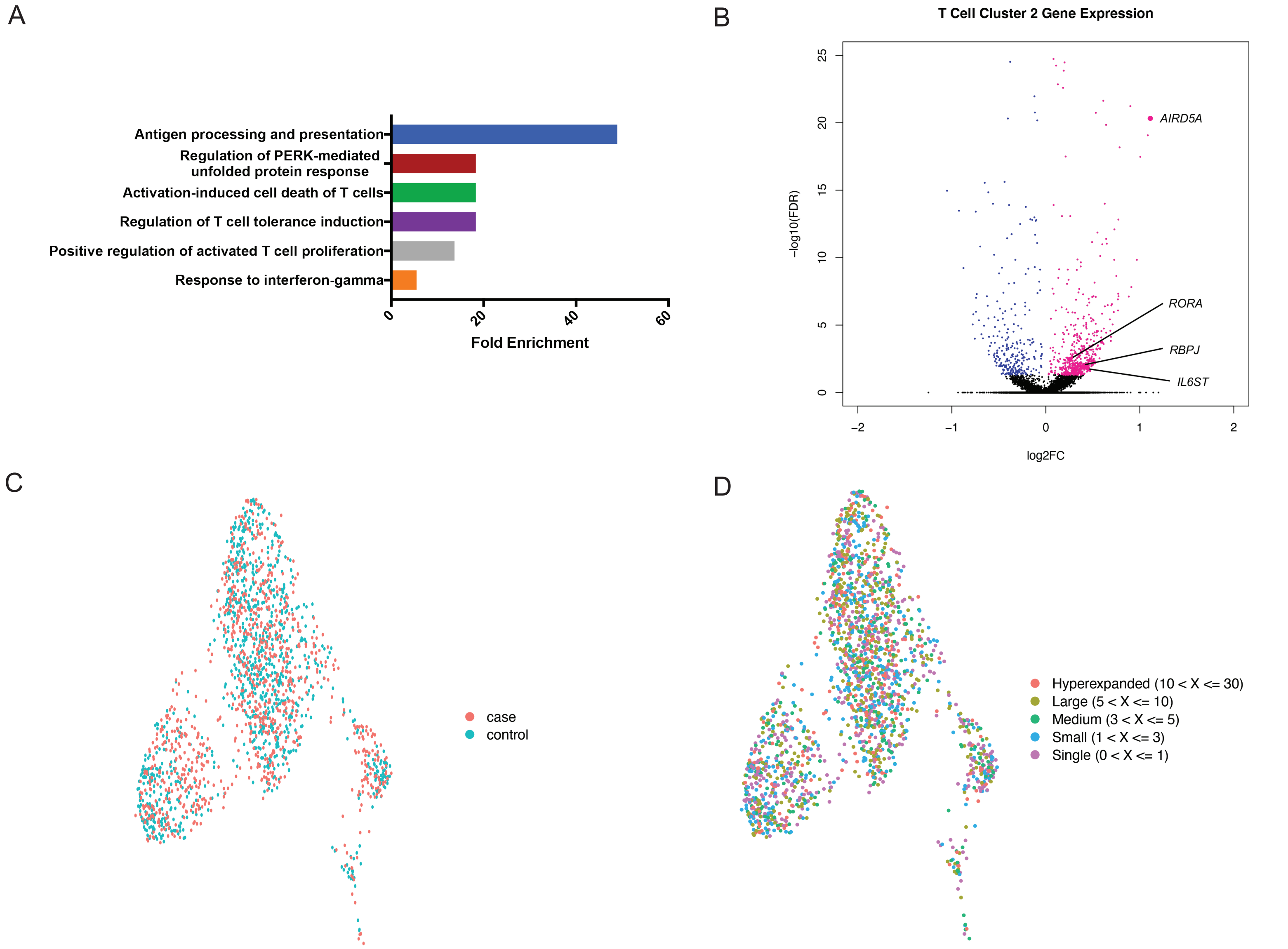

SUPPLEMENTAL FIGURE 3:
A. Select Gene Ontology (GO; Biological Pathway) results for differentially expressed genes between cases and controls in T cells. GO categories have FDR<0.05. X-axis indicates fold enrichment relative to the reference gene set of all expressed genes in the dataset. For a complete list of GO results, see Supplemental Table 1.
B. Volcano plot of DGE between cases and controls in T cell Cluster 2. Colored dots indicate statistical significance (FDR<0.05). Positive log2FC (pink dots) indicates higher expression in cases, and negative log2FC (blue dots) indicates higher expression in controls. N=3 samples per group.
C. UMAP displaying the distribution of case and control cells across three T cell (CD3D+) clusters.
D. UMAP displaying the distribution of clone types across three T cell (CD3D+) clusters.
