# Supplementary material for: Single-cell immunophenotyping of the fetal immune response to maternal SARS-CoV-2 infection in late gestation
Source: Res Sq. 2021 Mar 16:rs.3.rs-311000. Preprint. [Version 1] doi: 10.21203/rs.3.rs-311000/v1 (PMC7987103; doi:10.21203/rs.3.rs-311000/v1)
Supplement: Supplement [file 8af7a03993e45b50b219aca8.pdf]

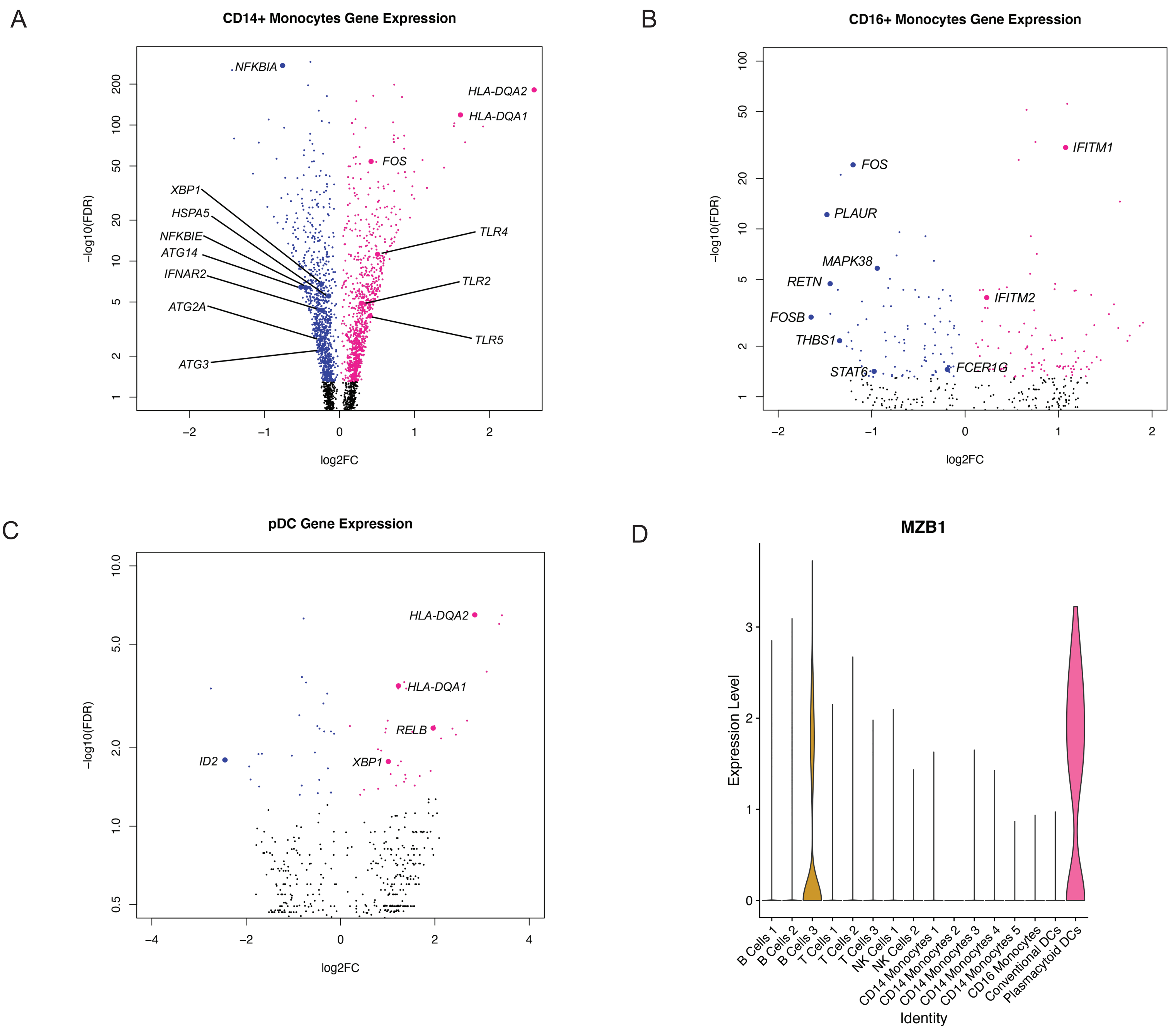

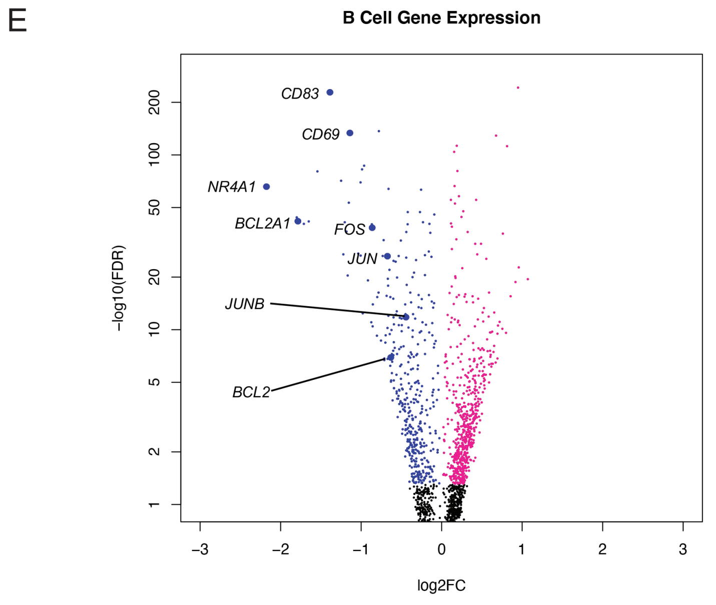

SUPPLEMENTAL FIGURE 2:
A. Volcano plot of DGE between cases and controls in CD14+ Monocytes. Colored dots indicate statistical significance (FDR<0.05). Positive log2FC (pink dots) indicates higher expression in cases, and negative log2FC (blue dots) indicates higher expression in controls. N=3 samples per group.
B. Volcano plot of DGE between cases and controls in CD16+ Monocytes. Colored dots indicate statistical significance (FDR<0.05). Positive log2FC (pink dots) indicates higher expression in cases, and negative log2FC (blue dots) indicates higher expression in controls. N=3 samples per group.
C. Volcano plot of DGE between cases and controls in pDCs. Colored dots indicate statistical significance (FDR<0.05). Positive log2FC (pink dots) indicates higher expression in cases, and negative log2FC (blue dots) indicates higher expression in controls. N=3 samples per group.
D. Violin plot of MZB1 expression across different cell clusters.
E. Volcano plot of DGE between cases and controls in B cells. Colored dots indicate statistical significance (FDR<0.05). Positive log2FC (pink dots) indicates higher expression in cases, and negative log2FC (blue dots) indicates higher expression in controls. N=3 samples per group.
