# Supplementary material for: Single-cell immunophenotyping of the fetal immune response to maternal SARS-CoV-2 infection in late gestation
Source: Res Sq. 2021 Mar 16:rs.3.rs-311000. Preprint. [Version 1] doi: 10.21203/rs.3.rs-311000/v1 (PMC7987103; doi:10.21203/rs.3.rs-311000/v1)
Supplement: Supplement [file 917e5e2ebbe363d60cb6cbfc.pdf]

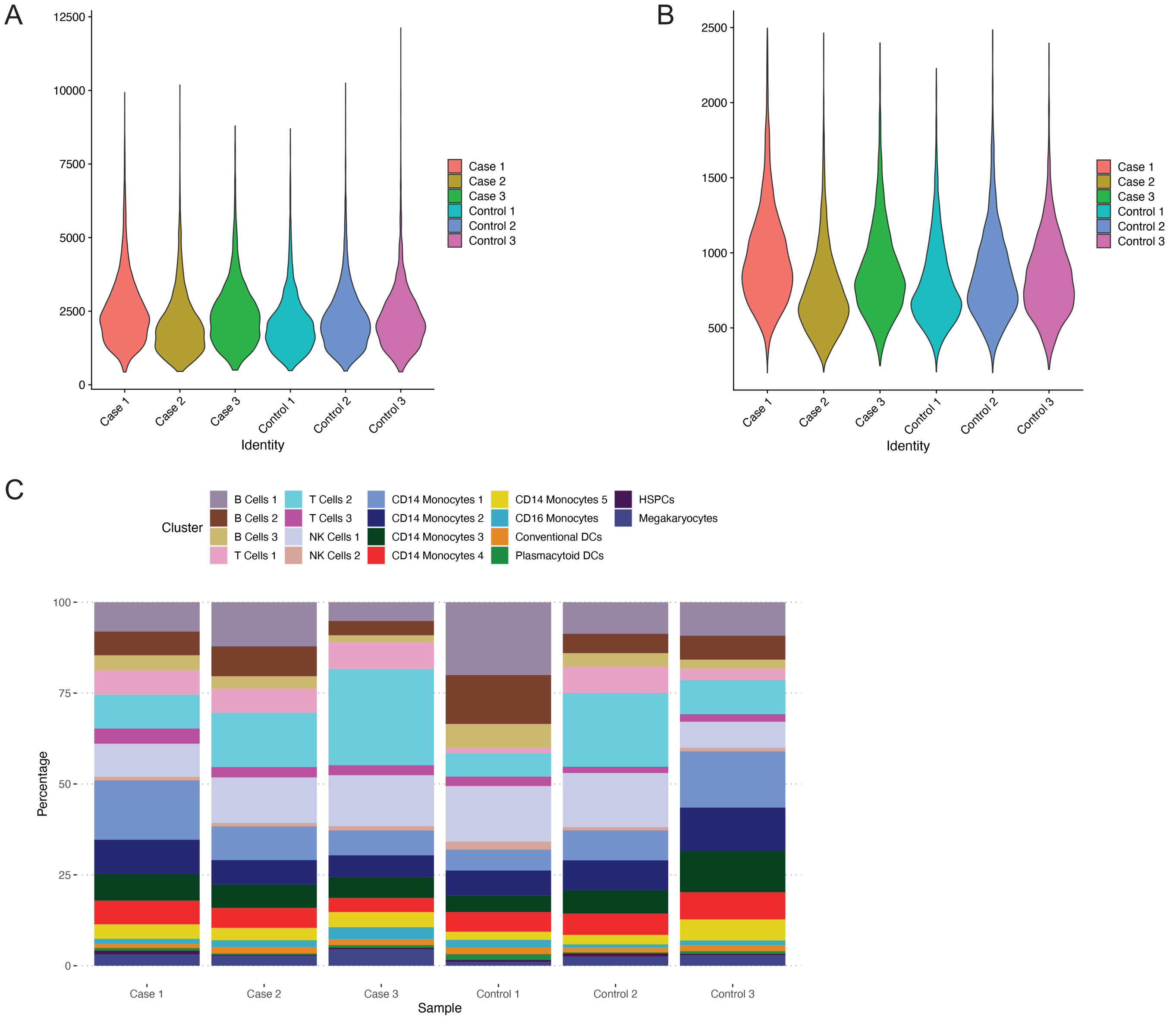

SUPPLEMENTAL FIGURE 1:
A. Violin plot of unique molecular identifiers (UMIs) detected per cell in each sample.
B. Violin plot of genes detected per cell in each sample.
C. Stacked bar plot of the percentage of cells within a given sample assigned to each cluster.
